# Post-Surgical Imaging Assessment in Rectal Cancer: Normal Findings and Complications

**DOI:** 10.3390/jcm12041489

**Published:** 2023-02-13

**Authors:** Federica De Muzio, Roberta Fusco, Carmen Cutolo, Giuliana Giacobbe, Federico Bruno, Pierpaolo Palumbo, Ginevra Danti, Giulia Grazzini, Federica Flammia, Alessandra Borgheresi, Andrea Agostini, Francesca Grassi, Andrea Giovagnoni, Vittorio Miele, Antonio Barile, Vincenza Granata

**Affiliations:** 1 Department of Medicine and Health Sciences V. Tiberio, University of Molise, 86100 Campobasso, Italy; 2 Medical Oncology Division, Igea SpA, 80013 Naples, Italy; 3 Department of Medicine, Surgery and Dentistry, University of Salerno, 84084 Salerno, Italy; 4 Division of Radiology, AORN Cardarelli, 80131 Naples, Italy; 5 Department of Diagnostic Imaging, Area of Cardiovascular and Interventional Imaging, Abruzzo Health Unit 1, 67100 L’Aquila, Italy; 6 Italian Society of Medical and Interventional Radiology (SIRM), SIRM Foundation, Via Della Signora 2, 20122 Milan, Italy; 7 Division of Radiology, Azienda Ospedaliera Universitaria Careggi, 50134 Florence, Italy; 8 Department of Clinical, Special and Dental Sciences, University Politecnica delle Marche, Via Conca 71, 60126 Ancona, Italy; 9 Department of Radiology, University Hospital “Azienda Ospedaliera Universitaria delle Marche”, Via Conca 71, 60126 Ancona, Italy; 10 Division of Radiology, Università degli Studi della Campania Luigi Vanvitelli, 80138 Naples, Italy; 11 Department of Applied Clinical Sciences and Biotechnology, University of L’Aquila, 67100 L’Aquila, Italy; 12 Division of Radiology, “Istituto Nazionale Tumori IRCCS Fondazione Pascale—IRCCS di Napoli”, 80131 Naples, Italy

**Keywords:** rectal cancer, normal findings and complications, surgery

## Abstract

Rectal cancer (RC) is one of the deadliest malignancies worldwide. Surgery is the most common treatment for RC, performed in 63.2% of patients. The type of surgical approach chosen aims to achieve maximum residual function with the lowest risk of recurrence. The selection is made by a multidisciplinary team that assesses the characteristics of the patient and the tumor. Total mesorectal excision (TME), including both low anterior resection (LAR) and abdominoperineal resection (APR), is still the standard of care for RC. Radical surgery is burdened by a 31% rate of major complications (Clavien–Dindo grade 3–4), such as anastomotic leaks and a risk of a permanent stoma. In recent years, less-invasive techniques, such as local excision, have been tested. These additional procedures could mitigate the morbidity of rectal resection, while providing acceptable oncologic results. The “watch and wait” approach is not a globally accepted model of care but encouraging results on selected groups of patients make it a promising strategy. In this plethora of treatments, the radiologist is called upon to distinguish a physiological from a pathological postoperative finding. The aim of this narrative review is to identify the main post-surgical complications and the most effective imaging techniques.

## 1. Introduction

Rectal cancer (RC) is one of the deadliest malignancies worldwide. According to GLOBOCAN 2021, the incidence and mortality of RC were 3.9% and 3.2%, respectively, with 732,210 new affected individuals worldwide each year [1].

Surgery is the most common treatment for RC, performed in 63.2% of patients [2]. Usually, surgical treatment is an upfront treatment for low-risk patients (T2 stage or lowest), while for high-risk patients this approach follows a neoadjuvant treatment (radiotherapy and adjuvant chemotherapy, as a long-course or short-course treatment) [3,4,5,6,7,8,9,10,11]. After neoadjuvant treatment, the type of surgical approach chosen aims to achieve maximum residual function with the lowest risk of recurrence, according to the location of the tumor and the degree of invasion of the pelvic floor and sphincter system [3].

Total mesorectal excision (TME) is the standard treatment for medium- and low-risk locally advanced rectal cancers (LARC) and has been shown to achieve 5-year local recurrence (LR) rates of only 2% to 10% [3]. TME records a perioperative mortality rate of 1–2%, which could increase in older patients with comorbidities, and it is also associated with a 31% rate of major complications (Clavien–Dindo grade 3–4), such as anastomotic leaks, a 25% risk of a permanent stoma, chronic altered bowel function, or anorectal and sexual dysfunction in more than 60% of patients [12]. Low anterior resection (LAR) is a sphincter-sparing TME technique. Although definitive colostomy could be avoided, up to 90% of patients following the procedure could experience disabling symptoms of the so-called LAR syndrome, that drastically reduce the quality of life [13,14]. Therefore, less-invasive strategies have been tested in order to mitigate the morbidity of rectal resection, while providing acceptable oncologic results, particularly for early-stage RC. In this context, organ-preserving treatment, such as local excision and "watch and wait", has become increasingly relevant [12,15,16,17]. Local excision is a minimally invasive approach that involves removal of only the tumor and the rectal wall layers [18]. Transanal endoscopic microsurgery (TEM) and transanal minimally invasive surgery (TAMIS) are recent excision techniques, which have been added to the surgeon’s armamentarium, improving visualization and access to the more proximal rectum [19,20,21,22].

Imaging plays a key role not only in the pre-operative phase, but also in the post-treatment period in assessing the response to neoadjuvant chemoradiotherapy (nCRT), postoperative complications, and disease recurrence [10,11,12]. Post-surgical anatomy could be difficult to interpret, and the radiologist should be familiar with the main surgical procedures and any associated pathological findings [2]. When a post-surgical complication is suspected, imaging allows to direct towards a conservative strategy or re-operation. Computed tomography (CT) with multiplanar reconstructions is the main method applied, with ultrasound (US) and magnetic resonance imaging (MRI) used in specific pathological conditions [2]. In particular, MRI is the main imaging modality employed for detecting and characterizing early recurrence of RC [23,24]. Early detection of LR is an important factor in avoiding disease progression to technical unresectability. Since recurrence is asymptomatic in 23–30% of cases, regular follow-up could improve early detection [25].

This narrative review aims to assess the most frequent surgical approaches used in the treatment of RC and the related main complications (anastomotic leak, hernia, and local recurrence) that could be detected on imaging.

## 2. Surgical Options

### 2.1. Radical Approach

TME is a core surgical technique in the treatment of RC [26,27]. TME is defined as an en block excision of the tumor with the mesorectum and all its contents along the planes of the mesorectal fascia (MRF) [26,27]. The main endpoint is to remove all foci of the disease by achieving a circumferential resection margin (CRM) of the surgical specimen [27,28]. TME includes both low anterior resection (LAR) and abdominoperineal resection (APR). LAR consists of a surgical resection with an intact anal canal and a coloanal anastomosis [27,28]. It is a technique applicable in tumors of the upper or middle rectum with an available margin of at least 5 cm from the anal border [29]. Low rectal tumors are treated with an ultra-low resection, consisting of a inter-sphincteric dissection and packing of a colo-anal anastomosis, with a possible J-pouch creation [30]. Extended LAR entails a resection of the proximal distal sigmoid colon, while high anterior resection prevents anastomosis of the sigmoid colon with the upper part of the rectum [30]. During the surgical procedure, mobilization of the splenic flexure is performed to avoid excessive pressure on the anastomosis, which justifies the presence of perisplenic fluid in the postoperative imaging evaluation [31].

APR is a more demolitive approach reserved for tumors that involve the anal canal, with the formation of an end colostomy [28,29,30]. Possible variants are the inter-sphincteric approach with intact external anal sphincter left in situ, and the extra-levator abdominoperineal excision (ELAPE) in case of invasion of the pelvic floor [28,29,30]. This destructive treatment leaves a large pelvic floor defect that could be treated with primary closure, placement of synthetic or xenograft mesh, or use of a myocutaneous flap [2].

Transanal TME is the latest minimally invasive procedure, combining abdominal TME with transanal endoscopic resection [32]. Thanks to the distal access, this technique would allow a better visualization under endoscopic guidance of the most challenging regions for proctectomy, being proposed as a preferable approach in the future for low rectal cancer resection [32,33].

On postoperative imaging, the colorectal anastomosis appears anterior to the presacral fascia and posterior to the prostate or vagina [2]. At the resection site, little fluid collection and gas are often present as physiological findings [31].

Pre-operative knowledge of the stage and the location of the tumor is indispensable in an upfront surgical approach, which should be reserved for early-stage tumors, preferably of the upper rectum to allow the preservation of the sphincter apparatus [27]. For early-stage tumors (T2N0) of the lower rectum, a surgical approach is reasonable in the first instance if anastomosis is possible [27,34]. In contrast, for LARC, surgical therapy could only be the next step after neoadjuvant induction therapy [17,34,35,36].

### 2.2. Local Excision

Local excision involves the removal of the tumor and a rectal margin to the perirectal fat, in the absence of proctectomy [18]. Different approaches are possible, and currently transanal excision (TAE) is the most applied [18]. Transanal endoscopic microsurgery (TEM) and minimally invasive transanal surgery (TAMIS) are the most recent techniques [37,38]. The difference between them concerns mostly the instrumentation used [37,38]. A full-thickness resection of the rectal lesion is performed, and the mural defect might be left to heal or closed with sutures [2].

On postoperative imaging, observable changes are minimal, such as air and mild inflammation of the mesorectum or a partial defect in the treated rectal wall [2]. Local excision should be considered as excisional biopsies that allow to evaluate the histopathological nature of the lesion, avoid a piecemeal biopsy, and estimate the need for a subsequent radicalizing intervention [37,38]. The main limitation is the possible risks of positive resection margins, proving a higher risk of locoregional recurrence and lower overall survival than TME [39]. Patient selection is crucial, and the European Society of Medical Oncology (ESMO) recommends local excision only for early-stage cancer (cT1N0) or for advanced T-stage with a high surgical risk [27].

### 2.3. Watch and Wait Strategy

**Figure 1:**
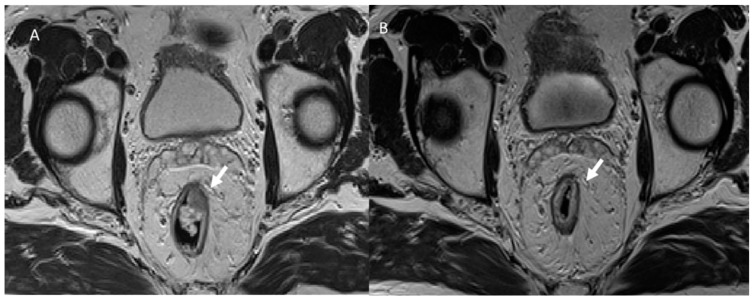
T2-W MRI assessment in pre (**A**) and post (**B**) neoadjuvant treatment for rectal cancer. In (**A**) pre-treatment, the arrow shows a T2 rectal tumor, with the radiological complete response in (**B**) (arrow).

RCs are very responsive to neoadjuvant therapy and may show highly satisfactory post-treatment results [12]. Starting from the observation of a complete pathological response (Figure 1), in some patients, alternative therapeutic approaches have been experimented that differ or that completely avoid surgery.

**Figure 2:**
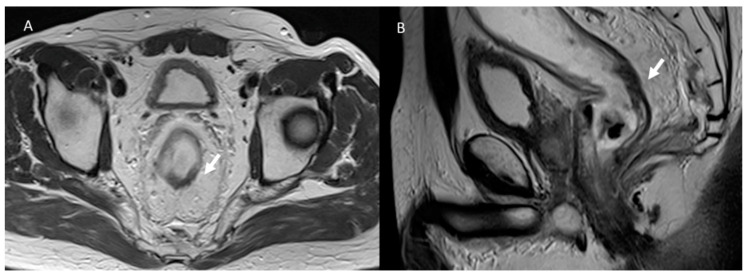
T2-W MRI assessment in post-treatment rectal cancer in the axial (**A**) and sagittal (**B**) plane. Arrows show fibrotic changes of the rectal wall without a residual lesion.

Habr-Gama et al. [40] sensed that the complete clinical response or fibrotic response (Figure 2) after neoadjuvant therapy could correspond to a pathological response, enlisting patients in monitoring programs for two years.

The results showed a similar oncological outcome between surgical and non-operative management [40,41,42]. Additional data have further confirmed these observations and support these initial results [43,44].

**Figure 3:**
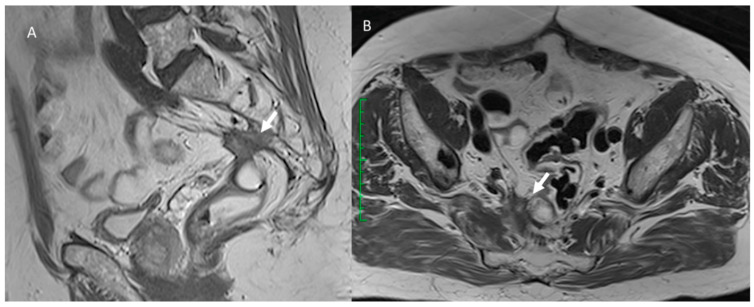
MRI assessment of rectal cancer relapse. In (**A**) (T2-W in axial plane) and (**B**) (T2-W in sagittal plane), arrows show relapse that involves rectal wall and external planes.

The International Watch and Wait Database (IWWD) reported 880 non-operative-managed patients in 2018 after complete clinical response to neoadjuvant therapy (47 centers in 15 European countries). This is the largest study and recorded that most relapses (Figure 3) occur within two years, mainly confined to the rectal wall. These results enable to safely apply a conservative strategy, taking into account endoscopic follow-up and the possibility of safely performing salvage surgery without any adverse oncological outcome from delayed definitive surgical resection [45].

Another viable strategy that requires the close collaboration of the multidisciplinary team is the so-called “intentional watch and wait”. In patients with low rectal cancer at an early stage (cT2N0), nCRT may be considered to allow an organ-preservation approach [42].

Currently, the “watch and wait” approach is not a globally accepted standard of care. Moreover, there is a lack of reference for patient selection and monitoring [46]. The most common protocol adopted suggests the combination of clinical assessment with digital rectal examination and endoscopy, with high-resolution MRI improving the assessment of complete clinical response [46].

Comprehensive risk–benefit evaluation and close collaboration between the different actors in the multidisciplinary team are fundamental prerequisites for making this therapeutic strategy safe and effective [47].

## 3. Common Postoperative Complications

### 3.1. Anastomotic Leak

**Figure 4:**
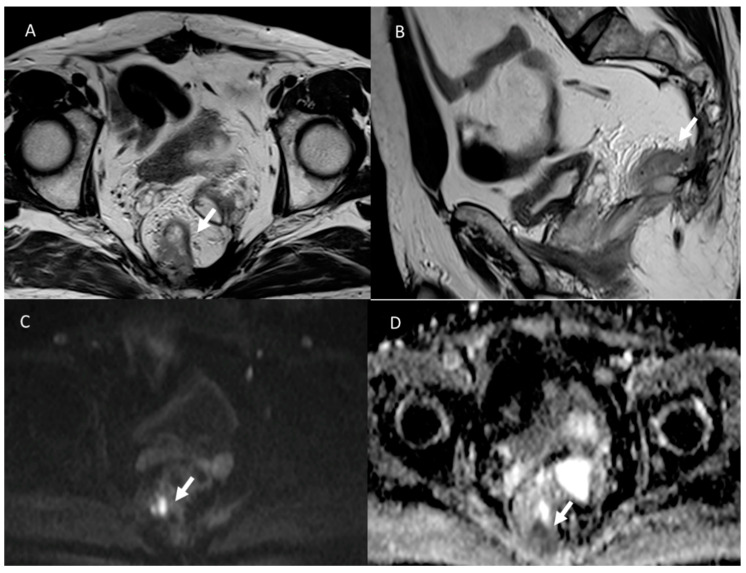
T2-W in sagittal plane (**A**) and T1-W post-contrast in sagittal plane (**B**) sequences of MRI assessment of post-surgical anastomotic leak. Arrows show a loss of continuity of the intestinal wall at the anastomosis site, with communication between the intra- and extra-luminal compartments. In DWI sequences (**C**: b800s/mm^2^ and **D**: ADC map), the lesion shows restricted diffusion.

Anastomotic leak (AL) is considered a major complication of colorectal surgery with an incidence between 1% and 19% [48] and a mortality rate of 10–15% [49]. According to the definition of the International Study Group of Rectal Cancer, the AL corresponds to a loss of continuity of the intestinal wall at the anastomosis site, with communication between the intra- (Figure 4) and extra-luminal compartments [50]. To standardize reporting of clinical studies, Rahbari et al. proposed a classification of AL into 1 of 3 grades (grade A, B, or C), depending on patients’ management [51]. Grade A is a mild form, involving only a possible delay of the ileostomy/colostomy closure. The management of patients with grade B requires an active therapeutic response, such as the administration of antibiotics or the placement of abdominal/pelvic drainage, while grade C corresponds to a severe clinical condition that needs an operational re-laparotomy [51].

ALs are also distinguished into early AL (within 30 postoperative days) and late AL (beyond 30 postoperative days or after discharge) [49]. Most ALs occur early in the postoperative period (between 5 and 10 days), with more severe clinical and radiological pictures of peritoneal contamination, compared to late forms that usually manifest with localized pelvic purulent collections [49,51]. The risk factors for early AL are correlated with the surgical technique, while the fragility of the patient seems to impact on late AL development [51,52]. A nCRT appears to increase the risk of late AL [52], due to the technical surgical complexity of the post-radiation pelvis and the delayed effect on tissue healing [53].

Another independent risk factor in the development of AL is the distance of the tumor from the anal verge. The two factors are inversely correlated, with a higher risk of AL in lower tumors [54]. Common symptoms and signs suggestive of AL are fever, intense abdominal pain, tachycardia, and abdominal tenderness [2]. However, AL could be completely asymptomatic, and the diagnosis could be complicated. CT with intravenous contrast medium (CECT) and endoluminal contrast medium is currently the preferred imaging technique for assessing the possible presence of an AL [55]. CT could confirm clinical leakage in 48–100% of cases [31]. The administration of endorectal contrast prior to CT scans and close evaluation with multiplanar reconstructions allow to identify the contrast extravasation through the wall defect [31]. Regardless of the surgical technique used, in the normal postoperative course, pneumoperitoneum resolves within 10 days (on average within the first 5 days) [2] and, although the presence of free air is a physiological finding after surgery, free intra-abdominal gas could be an indirect sign of an AL [2,31]. The persistence of the free air beyond this period and in particular beyond six months after surgery is highly suggestive of a leak [2,31]. It should be noted that the sensitivity of the CT in the detection of AL is relatively low (65%), and the possibility of reintervention should always be evaluated if the clinical suspicion of AL persists [56,57,58,59]. The detection of perianastomotic fluid and purulent collections are other ancillary radiological features [31]. Some authors suggest considering a pelvic abscess at the surgical site as a direct sign of AL [51]. As in other locations, an abscess appears as a collection with a hypodense necrotic core at relatively low attenuation values (10 to 30 HU) and inflammatory enhanced walls, showing obliteration of adjacent fascial planes in advanced stages of infection spread [31]. In order to achieve a conservative management, a US- or CT-guided drainage should be the therapy of choice, along with antibiotic administration, reserving more invasive approaches only for refractory cases [56,57].

### 3.2. Fistula

**Figure 5:**
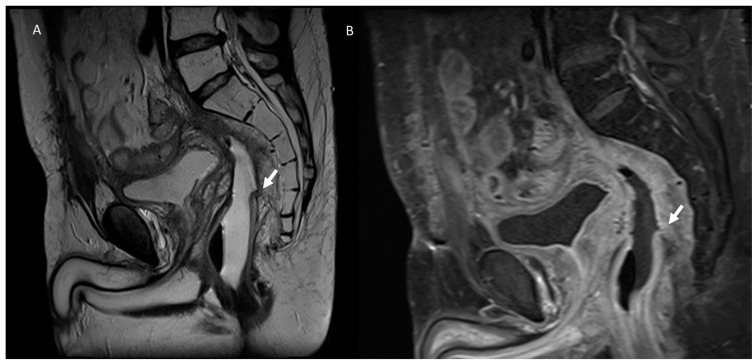
T2-W in sagittal plane (**A**) and T2-W in axial plane (**B**) MRI assessment of post-surgical fistula with sacral plane (arrows).

Continuous leakage leads to chronic inflammatory processes, culminating in a fistula formation. Although fluoroscopic fistulography and water-soluble contrast medium enema (WSCE) are able to clearly show the pathological communication between adjacent tissues, they lack the panoramic and contrast resolution of cross-sectional imaging [2]. CECT with endoluminal contrast agent could be useful in the definition of perineal and perianal fistula and may highlight pelvic collections within contiguous structures [60]. However, MRI is definitely the gold standard in the assessment of pelvic fistulas [61]. MRI could reveal complexity and multiplicity of a fistula and the stage of progress [62]. Hyperintensity in T2-weighted sequences and an avid contrast-enhancement characterize a fistula in the active phase, whereas a fibrotic tract tends to be less hyperintense in T2-weighted images (Figure 5) [61,62].

Diffusion-weighted sequences could refine the evaluation of the extent and the external or internal openings of fistulas [62]. In addition, gadolinium administration may be useful in the evaluation of wall inflammation and surrounding fat planes [61,62].

### 3.3. Bleeding

Postoperative bleeding is a rare but potentially fatal complication. The overall risk is around 6%, and in 1% of cases it may manifest as massive hemorrhage and hemodynamic instability [63,64]. During surgery, a presacral fascia injury or avulsion of the rectosacral fascia could damage the presacral and basivertebral veins, causing bleeding that is difficult to manage with conventional hemostatic maneuvers [65]. Presacral venous plexus damage occurs more often in patients with advanced tumors or in the case of “hostile pelvis”, caused by radiotherapy [2]. When the clinical and laboratory data suggest postoperative bleeding, a multiphasic CECT should be performed [2]. CECT could quickly intercept the site of leakage and distinguish into active arterial extravasation during arterial phase scans and venous bleeding in the portal and late phases [2].

### 3.4. Urological Injury

Iatrogenic genitourinary tract lesions are rare events (0.71%), that are associated with advanced disease or anatomical changes due to previous radiotherapy [66]. Ureteral lesions are particularly insidious, as they are often misdiagnosed intraoperatively. Hematuria is the most common symptom, which may be completely absent in 15–45% of all injuries [67].

Regarding diagnostic management, US may be the first tool that should be utilized, showing hydronephrosis, the absence of ipsilateral ureteral jet, and ascites [68]. CT urography in the excretory phase is certainly more accurate in directly identifying any ureteral or bladder lesions, showing contrast medium extravasation associated with possible collection in the retroperitoneal or peritoneal space [69,70].

The urethra may be damaged during operative maneuvers in APR. In these circumstances, a water-soluble contrast medium urethrogram could depict contrast medium spillage through the wall lesion [69].

### 3.5. Wound Infections

Wound infections occur in 5–10% of patients undergoing major abdominal surgery [31]. The infectious process could lead to important sequelae, such as wound dehiscence, peritonitis with sepsis, and hernia formation [31].

Perineal resections in APR are most often associated with varying degrees of wound complications, from minor dehiscence to fistula and perineal hernia formation. Treatment options depend on the severity of the clinical picture, ranging from antibiotic therapy to incision or drainage of infected collections [31]. In the case of wound dehiscence, CT may show the presence of fluid collections with air within the wound or in adjacent tissues [31].

### 3.6. Hernia

Incisional hernia is a relatively frequent complication in abdominal surgery, with a prevalence of 0.5–50%, more commonly observed after vertical incisions [71,72]. Stomal or parastomal herniation of mesenteric fat or bowel is a frequently expected finding, which does not entail substantial consequences unless complicated by bowel obstruction, occurring in 3.8 × 10^7^% of cases [71,72].

**Figure 6:**
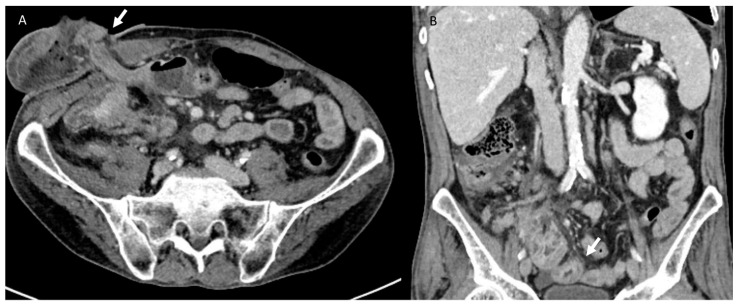
CT assessment of stomal hernia. In (**A**) (axial plane), the arrow shows parastomal fat accumulation at the stoma site. In (**B**) (MPR coronal plane), the arrow shows intestinal wall edema.

CT findings that suggest progression to an impending complication are significant wall edema, parastomal fat stranding, and fluid accumulation at the stoma site (Figure 6). Synthetic placement of a prophylactic mesh has been shown to be effective in reducing parastomal hernia rates [73].

A protrusion of a viscera through a defect in the peritoneal or retroperitoneal compartments configures the potentially life-threatening condition of internal hernia [73]. Although the reported rates only reach 0.65% [74], the main concern is vascular impairment with intestinal ischemia and subsequent necrosis. On CT scans, an internal hernia could be recognized by a sac-like cluster of dilated small bowel loops [75]. A transition zone between the proximal dilated bowel loops and the normal or collapsed distal bowel could be identified [75]. Other characteristic signs are the crowded and stretched appearance of the vascular pedicle of the herniated loops and the whirlpool sign, i.e., the whirlpool appearance of the mesenteric vessels at the twisting point [74]. In cases of strangulation, wall thickening and hippo-enhancement, pneumatosis, and ascites may be seen, suggesting ischemia [76].

Perineal hernias may occur in up to 30% of APRs. In particular, ELAPE is associated with a higher incidence of wound complications than conventional APR. A closed-loop small bowel obstruction with gangrene is an extremely rare event that can occur up to 7 years after ELAPE [77].

### 3.7. Local Recurrence

Local recurrence is a common complication in RC patients [78,79].

The introduction of TME and neoadjuvant therapy has significantly reduced the LR rate from 30% to 10% [78,79]. Approximately 80% of disease relapses could arise within 3 years after surgery, and 95% within 5 years (1–2). Follow-up intervals (4–6 months for the first 3 years, 6 months in the following 2 years) and the overall duration (5 years) have been defined on the basis of this evidence. This management is also supported by a retrospective analysis on a large sample of more than 20,000 patients, which stressed the life expectancy of patients treated for non-metastatic colorectal neoplasm in the absence of recurrence, completely overlapping on the general population after 5 years [80,81].

Societal recommendations identify CT as the imaging method of choice in the evaluation of patients after TME, while MRI plays a problem-solving role in unclear cases [46]. MRI examination is recommended in the re-evaluation of patients after neoadjuvant therapy who are candidates for a “watch and wait” strategy and in those who have undergone to transanal resection of RC [46].

The most important prognostic factor in case of LR is the possibility of obtaining a free resection margin (R0) after surgical treatment. This circumstance only occurs in 60% of cases [82], and the site of recurrence is significant in this regard. According to reports from the Mayo Clinic and the Memorial Sloan-Kettering Cancer Center (MSKCC) [83,84], the LR sites could be classified into four categories: (1) Axial (or central): includes anastomotic recurrence after low anterior resection, local recurrence after transanal or trans-sphincteric excision, and perineal recurrence after abdominoperineal resection. (2) Anterior: with recurrence in the bladder, vagina, uterus, seminal vesicles, or prostate. (3) Posterior: involving the sacrum and coccyx, or sacral root sheaths. (4) Lateral: involving the bony pelvic sidewall or sidewall structures, including the iliac vessels, pelvic ureters, lateral lymph nodes, pelvic autonomic nerves, and sidewall musculature [83,84,85]. The MSKCC group reported that recurrences in the lateral compartment are associated with a reduced rate of complete resectability (R0) compared to other locations [83,84].

**Figure 7:**
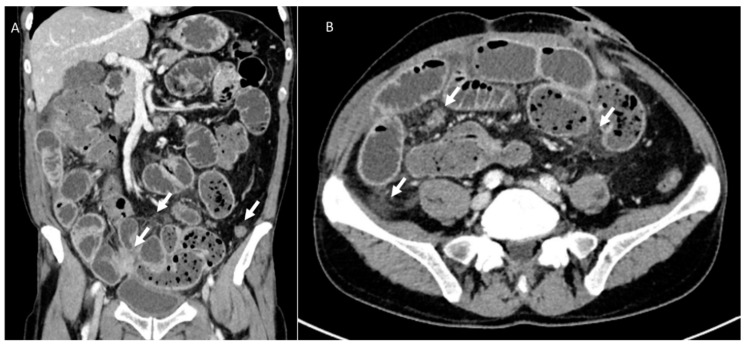
CT assessment during follow-up of rectal surgical-treated patient. In the MPR coronal plane (**A**) and axial plane (**B**), arrows show peritoneal carcinosis with intestinal occlusion.

The possibility of pelvic sidewall recrudescence correlates with tumor location, with higher rates for lower tumors [83,84]. This phenomenon could be related to the different lymphatic drainage of lower rectal cancer, with the increased possibility of residual micro-metastatic foci in the pelvic sidewall [86]. Hence, an accurate imaging technique that could clearly identify the stage of the disease and direct the patient to the tailored therapy is essential. CT is a widely used method in the surveillance of patients treated for RC. On CT, a suspected malignant area could appear as a nodule of soft tissue with inhomogeneous contrast enhancement that is newly arising or progressively growing [46] (Figure 7). The sensitivity and specificity of CT in detecting LR are between 82–91% and 69–72%, respectively [25].

MRI could achieve a considerably higher rate of sensitivity (80–90%) and specificity (near 100%) [25]. MRI could also appreciate tumor invasion through the assessment of the obliteration of fat planes between the rectum and adjacent organs [74]. Malignant tissue could appear as an area of high signal intensity with irregular margins in morphological T2-weighted images [87]. However, the same behavior could be recorded in the case of hematoma, granulation tissue, and radiotherapy-induced inflammatory changes that may persist for more than two years after treatment [87]. Another challenging condition on morphological sequences is the distinction between the simple fibrotic response and scar tissue hiding tumor cell clusters [87]. Increasing evidence suggests that diffusion-weighted (DW) sequences may be more effective in enabling this differentiation. Tumor foci could show high signals in DW sequences at high b-values, while fibrotic tissue has a very short *T*_2_ relaxation time that results in low signals in both the apparent diffusion coefficient (ADC) map and DW images [88,89]. Differences in recorded ADC values could also help distinguish scar tissue from tumor recurrence after surgical resection [90], the latter having significantly lower values with respect to inflammatory changes [91,92]. The added value of diffusion during the post-treatment follow-up period is well-documented.

Reports focusing on patients with organ-preservation treatment (chemoradiotherapy + transanal endoscopic microsurgery, or “watch and wait”) have shown an increased sensitivity of MRI in the diagnosis of tumor regrowth, allowing the detection of small pathological nodules, even before morphological sequences and endoscopy [93,94].

In disseminated disease, the tumor nest prominently stands out against the dark background in DW images, capturing the radiologist’s attention generally focused more on the rectal and pararectal areas [95].

The use of gadolinium does not appear to be of significant benefit in the assessment of disease recurrence. Molinelli et al. observed that the integration of the post-contrast T1 FS 3D sequences increased the sensitivity for each of the three readers involved (94.4% for all readers) in their study, but the specificity values resulted as substantially increased only in less experienced readers (reader G: 96%, reader E: 84%, reader V: 80%) [78]. Moreover, regarding AUC values obtained from the comparison of T2 + post-contrast T1 sequences and T2 + DWI images, researchers reached no statistically significant difference, as it has been shown in previous reports [78,95]. The same considerations could be applied to dynamic contrast-enhanced magnetic resonance imaging (DCE-MRI), which has shown no overall added value based on AUC over standard MRI (T2-weighted imaging and DWI) to identify complete responders to chemoradiotherapy [87,96]. Furthermore, the use of contrast not only carries the risk of adverse reactions, but also entails additional costs and time to complete the examination [87,97].

A recent study evaluated the possibility of applying tissue models in distinguishing between anastomotic recurrence and benign tissue, which is a major problem in post-treatment follow-up. The researchers showed that all models based on morphological and functional sequences showed good diagnostic performance, with AUCs from 0.719 to 0.864, with higher diagnostic performance of the combined model compared to all individual ones [98]. This could be attributed to the interaction of the features of different MRI sequences reflecting tissue characteristics from distinct dimensions, overcoming the limitations of a single sequence [98,99,100,101]. Based on these results, it may be possible to perform biopsies only when strictly necessary.

Several reports have described the role of radiomics as a precision medicine tool [102,103], that could influence therapeutic approaches in RC [104,105,106,107,108,109,110,111,112,113,114,115]. Recently, the idea that imaging studies contain a great quantity of data, in the form of grey-level patterns, which are imperceptible to the human eyes, has become more and more interesting [116,117,118,119,120,121,122,123,124,125,126,127,128,129,130,131,132,133,134]. These texture features, when correlated with clinical-pathological data and outcomes [135,136,137,138,139,140,141,142,143,144,145,146,147,148,149,150,151,152,153], theoretically allow diagnostic and prognostic assessment [154,155,156,157,158,159,160,161,162]. The assessment of textural characteristics, obtained by radiological images, which depend on mathematical analysis, such as histogram analysis, is called radiomics [163,164,165,166,167,168,169,170,171,172,173,174,175,176]. This approach is captivating since it should allow to extract biological data from the radiological images [177,178,179,180,181,182,183,184,185,186,187,188,189,190,191,192,193,194,195,196,197] without an invasive approach, reducing costs and time and avoiding any risk for the patients. For several tumors, radiomic analyses have already provided an accurate evaluation of biology, allowing the identification of indices correlated with clinical outcomes [184,185,186,187,188,189,190,191,192]. Imaging features and texture analysis (TA) built on MR images proved to be able to intercept the therapeutic response to nCRT and tumor recurrence in patients with LARC [111,112,113,114,115]. These preliminary data suggest that it is possible to accurately select patients, building customized, increasingly organ-sparing, diagnostic and therapeutic pathways.

## 4. Discussion

The knowledge of the main type of surgical procedure for rectal cancer patients is necessary for the radiologist to recognize the common radiological features of postoperative findings and identify the possible postoperative complications, such as the tumor recurrence, as soon as possible to improve the outcome. In this scenario, it is clear that it is also crucial to choose the most suitable diagnostic tool and to optimize the study protocol.

Regarding the “watch and wait approach”, the adverse event is linked to a regrowth of the lesion in a patient who had a complete clinical and radiological response. Therefore, the main tool to use is MRI, with restricted follow-up intervals (3 months), and a study protocol that includes T2-W sequences and DWI, while T1-W contrast sequences are optional. Specific parameters should be followed to achieve optimal high-resolution sequences, including a small FOV, a small slice thickness (no more than 3 mm), and the correct scan plane alignment (perpendicular to the rectal wall at the level of the tumor). On T2-W sequences, the recurrence showed the same findings as pre-treated lesions: a solid hyperintense lesion, with or without a more intense area if it is a mucinous lesion, and a restricted signal on DWI sequences [40,198,199].

Regarding minimally invasive procedures, the radiological management is more complicated and is correlated either with complications’ detection or tumor regrowth. Since complications are procedural, that is due to the surgical approach, they occur early and are confined to the pelvis or rectal wall. Frequently, we can find a fistula or the interruption of the continuity of the rectal wall, and less commonly an abscess (if the previous ones are not diagnosed early) or bleeding. Therefore, the diagnostic tool should be chosen according to the clinical aspects. MRI is preferred for fistulas, abscesses, and rectal wall lesions, although the CT with endorectal contrast medium also allows a proper diagnosis [40]. Instead, for bleeding, the multiphase contrast study CT remains the main diagnostic tool. With regard to recurrences, for local regrowth, MRI is the main tool and should be combined with total body CT to assess extra-pelvic localization (e.g., lungs, liver). The follow-up timing should be as proposed by international guidelines or in research settings according to the procedure [27].

Regarding TME, since this approach is more complicated with respect to minimally invasive procedures, there are different scenarios that should be considered.

For the LAR approach, it is possible that there are two different surgical times: the first, in which there are the lesions and mesorectal surgical resection, and the second, in which a coloanal anastomosis is performed. This approach should allow to avoid an anastomotic leak. Therefore, the radiologist’s role in using MRI is critical since it is necessary to assess the rectal wall status (edema due to radiotherapy treatment and vascularization status). Consequently, contrast medium should be employed [198,199].

With regard to the APR approach, as for LAR patients after the anastomosis phase, the diagnostic management of early or late complications is the same as the one employed for minimally invasive procedures [27].

In this context, it is evident that the properly treated rectal cancer patient management requires a multidisciplinary (radiologist, interventional radiologist, surgeon, oncologist) and multimodality (MRI, CT, endoscopy) approach.

## 5. Conclusions

Surgery is the most common treatment for rectal cancer. In recent decades, there has been a shift from a more invasive approach to organ-preserving strategies, according to patient and tumor features. The radiologist is required to know the different surgical treatments and probable complications in order to guide the patient to the correct management. CT is the primary modality of choice in most cases, allowing for the early detection of major complications, such as anastomotic leakage and internal hernia, which could hesitate in the acute abdomen.

Through careful patient selection, the “watch and wait” approach could be applied as long as there is close monitoring for disease recurrence. MRI allows precise detection of local relapses, albeit some physiological changes after surgical and radiotherapy treatment could be difficult to interpret.

Radiomics appears to be promising in this field of application and new evidence is needed to support the preliminary data.

## Data Availability

Data are reported in the manuscript.
